# Association between Latest Activated Sites in the Left Ventricle and Akinetic Segments in Patients with Ischemic Cardiomyopathy

**Published:** 2016-07-06

**Authors:** Hakimeh Sadeghian, Aliasghar Kousari, Shahla Majidi, Mehrnaz Rezvanfard, Ali Kazemisaeid, Seyed Ali Moezzi, Ali Vasheghani Farahani, Morteza Abdar Esfahani, Mohammad Sahebjam, Arezoo Zoroufian, Afsaneh Sadeghian

**Affiliations:** 1 *Tehran Heart Center, Tehran University of Medical Sciences, Tehran, Iran.*; 2 *Emam Khomeini Hospital, Ahvaz University of Medical Sciences, Ahvaz, Iran. *; 3 *Isfahan University of Medical Sciences, Isfahan, Iran.*; 4 *Shahroud University of Medical Sciences, Shahroud, Iran.*

**Keywords:** *Cardiomyopathies*, *Ischemia*, *Heart ventricles*

## Abstract

**Background: **It is not clear whether the latest activation sites in the left ventricle (LV) are matched with infracted regions in patients with ischemic cardiomyopathy (ICM). We aimed to investigate whether the latest activation sites in the LV are in agreement with the region of akinesia in patients with ICM.

**Methods: **Data were analyzed in 106 patients (age = 60.5 ± 12.1 y, male = 88.7%) with ICM (ejection fraction ≤ 35%) who were refractory to pharmacological therapy and were referred to the echocardiography department for an evaluation of the feasibility of cardiac resynchronization therapy. Wall motion abnormalities, time to peak systolic myocardial velocity (Ts) of 6 basal and 6 mid-portion segments of the LV, and 4 frequently used dyssynchrony indices were measured using 2-dimensional echocardiography and tissue Doppler imaging (TDI). To evaluate the influence of the electrocardiographic pattern, we categorized the patients into 2 groups: patients with QRS ≤ 120 ms and those with QRS >120 ms.

**Results: **A total of 1 272 segments were studied. The latest activation sites (with longest Ts) were most frequently located in the mid-anterior (n = 32, 30.2%) and basal-anterior segments (n = 29, 27.4%), while the most common sites of akinesia were the mid-anteroseptal (n = 65, 61.3%) and mid-septal (n = 51, 48.1%) segments. Generally, no significant concordance was found between the latest activated segments and akinesia either in all the patients or in the QRS groups. Detailed analysis within the segments indicated a good agreement between akinesia and delayed activation in the basal-lateral segment solely in the patients with QRS duration ≤ 120 ms (Φ = 0.707; p value ≤ 0.001).

**Conclusion:** The akinetic segment on 2-dimensional echocardiogram was not matched with the latest activation sites in the LV determined by TDI in patients with ICM.

## Introduction

Heart failure is a debilitating poor-prognosis disease characterized by progressive left ventricular (LV) dilation and loss of contractile function.^     1 ^ LV dyssynchrony and wall motion abnormalities are 2 frequent features in patients with heart failure, which may exacerbate cardiac dysfunction in these patients.^2^^-^^4^ 

Cardiac resynchronization therapy (CRT) is a rapidly evolving and promising treatment option in drug-refractory heart failure patients with a depressed LV systolic function.^5^^, ^6 A quantitative evaluation of LV dyssynchrony is reliably conducted using tissue Doppler imaging (TDI) in CRT candidates^7^^-^^10^ and helps investigators achieve a more desirable response through optimal lead placement in the latest activated sites.^11^^-^^13^ There are reports indicating that infracted regions have slower conduction velocity than corresponding areas in normal subjects^14^^, ^^15^ and lead placement in the latest activation areas leads to optimal results.^12^^, ^^13^ On the other hand, leads positioned in a scar tissue lead to a poor outcome in patients with heart failure.^16^^, ^^17^ The question is, therefore, whether these sites of latest activation are matched with infracted regions. Segmental akinesia is a sensitive indicator of transmural infarction,^   18 ^ and wall motion evaluation is regularly conducted via conventional echocardiography prior to CRT in patients with heart failure. Thus, in the current study, we sought to investigate whether there is any concordance between akinetic LV segment/s and latest activated site/s in patients with advanced heart failure secondary to ischemia.

## Methods

 From January 2006 to December 2009, a retrospective review was conducted of the data on 106 patients with advanced heart failure secondary to ischemia who were referred to one of the echocardiography clinics in Tehran Heart Center, Tehran, Iran, for echocardiographic evaluation. All the patients had severe heart failure (New York Heart Association [NYHA] functional class ≥ III) and left ventricular ejection fraction (LVEF) ≤ 35%, which was refractory to optimized drug therapy. Patients with a history of recent (< 3 mon) unstable angina or myocardial infarction, valvular heart disease, or previous pacemaker implantation were excluded from the study. Ischemia was diagnosed as the cause of heart failure if the patients had a history of myocardial infarction or revascularization or angiographic evidence of stenosis > 75% of the left main or the proximal left anterior descending artery, or stenosis > 75% of 2 or more epicardial vessels.^   19 ^ All the patients underwent complete resting transthoracic echocardiography and TDI for the evaluation of regional wall motion and the extent of LV dyssynchrony as well as determination of the site of the latest mechanical activation.

A combination of transthoracic 2-dimensional echocardiography, M-mode, pulsed, and continuous-wave Doppler with color-flow imaging was performed using a commercially available ultrasonographic system (VIVID 7, Vingmed GE, Horten, Norway with a 3.5-MHz transducer). All the following parameters were measured according to the guidelines of the American Society of Echocardiography^   20 ^: right and left ventricular dimensions (VD), left ventricular end-systolic volume (LVESV), left ventricular end-diastolic volume (LVEDV), pulmonary artery pressure, and tricuspid annular plane systolic excursion (TAPSE). Additionally, LVEF was evaluated using the eyeball method and the Simpson method via a multiplane modality of a 4-dimensional probe. From a currently used grading system, the severity of mitral regurgitation was scored 1 as mild, 2 as moderate, 3 as moderate to severe, and 4 as severe^21^ and tricuspid regurgitation was graded 1 as mild, 2 as moderate, and 3 as severe.^   22 ^

The opening and closing times of the aortic and pulmonic valves were also measured using the systolic blood flow by pulsed Doppler, with the sample volume placed at the level of the aortic and pulmonic annulus. Aortic pre-ejection time was measured from the beginning of the QRS complex to the beginning of the aortic flow velocity curve recorded by pulsed-wave Doppler in the apical view. Likewise, pulmonary pre-ejection time was measured from the beginning of the QRS complex to the beginning of the pulmonary flow velocity curve recorded in the left parasternal short-axis view. The difference between the 2 values determined interventricular mechanical delay (IVMD). A cut-off value = 40 ms was considered for interventricular dyssynchrony.^   23 ^

2D echocardiography is a safe and simple noninvasive technique to quantify LV regional wall motion. For a semiquantitative analysis of regional wall motion, a 12-segment model was employed that included the septal, anteroseptal, anterior, lateral, inferior, and posterior segments at the basal and mid-levels of the LV, respectively. All these segments were ranked based on the degree of both wall thickening and endocardial motion abnormalities as follows: normal (normal myocardial inward motion and wall thickening), hypokinetic (marked reduction in endocardial motion and wall thickening), akinetic (virtual absence of inward motion and wall thickening), and aneurismal (paradoxical wall motion away from the center of the LV in systole).^24^^, ^^25^

**Figure 1:**
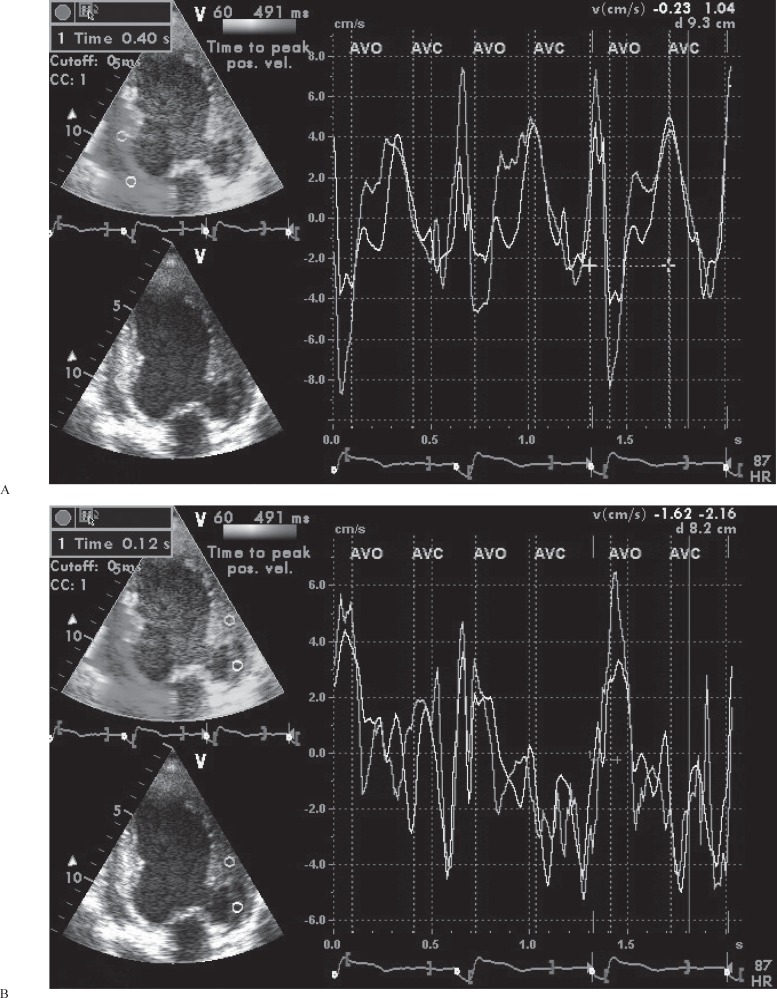
Measurement of time to peak systolic velocity of A) the inferior basal and mid-portion segments and B the) anterior basal and mid-portion segments.

Systolic synchronicity was assessed via TDI using the apical views (4-chamber, 2-chamber, and long-axis views) of the LV as previously described.^   26 ^ With adjustments of the filter frequency, gain settings, pulse repetition frequency, and color saturation, at least 3 consecutive beats were stored and the images were digitally stored for off-line analysis. Twelve segments (6 basal and 6 mid), similar to those evaluated by 2-dimensional echocardiography, were analyzed. The timing of the systolic events was evaluated by measuring the time to peak systolic myocardial velocity (Ts) in each LV segment utilizing the onset of the QRS complex as the reference point (Figure 1). The latest LV segment/s was defined as the segment/s with the longest Ts duration among the 12 LV segments. The following 4 parameters of systolic asynchrony were computed. The predictive cut-off values for a positive response to CRT are shown in parentheses according to previously reported data. *All segments delay (Ts-all-delay*): Differences between the longest and shortest Ts in 12 LV segments (105 ms).^   27^ *Basal segments delay (Ts-bas-delay)*: Differences between the longest and shortest Ts in 6 LV basal segments (78 ms).^   27 ^*All segments standard deviation (Ts-all-SD)*: SD of Ts of all the 12 LV segments (34.4 ms).^   27^ *Basal segments standard deviation (Ts-bas-SD)*: SD of Ts of 6 LV basal segments (34.5 ms).^   27 ^

According to our previous study, inter- and intraobserver variability for time to peak systolic velocity was 13 ± 11% and 18 ± 20% at our institution, respectively.^   26 ^

The data are presented as means ± SDs for the numerical variables. The categorical variables are expressed in terms of absolute frequencies and percentages. The agreement between the regions with longest Ts and the presence of akinesia was computed using the phi (Φ) coefficient in various LV segments. To investigate the effect of the QRS duration on the concordance between the akinetic LV segment/s and the latest activated site/s, we performed additional analysis comparing the patients with QRS duration ≤ 120 ms and QRS duration > 120 ms. Probability values ≤ 0.05 were considered statistically significant. For the statistical analyses, the statistical software SPSS, version 13.0, for Windows (SPSS Inc., Chicago, IL) was used. 

## Results

The patients’ demographic data, clinical characteristics, pharmacological medication, and detailed conventional echocardiographic findings are summarized in Table 1 while TDI measurements in all the patients and also in the QRS groups (> 120 ms and ≤ 120 ms) are summarized in Table 2. A total of 1 272 segments were technically adequate for the evaluation of regional activation delay. Regional Ts were significantly associated with the QRS duration and was significantly more delayed or tended to be more delayed in the patients with a wide QRS duration than in those with a narrow QRS duration in all the LV segments. Similarly, the frequency of interventricular dyssynchrony was significantly higher in the patients with a wide QRS duration than in those with a narrow QRS duration: 30 (38.0%) in the wide QRS group vs. 3 (11.0%) in the narrow QRS group. In regard to intraventricular dyssynchrony indices, the frequency of the intraventricular dyssynchrony markers varied from a minimum of 49.1% (53 cases), as defined by the Ts-all-delay dyssynchrony index, to a maximum of 53.8% (58 cases), as defined by the Ts-bas-delay dyssynchrony index, in all the patients. Intraventricular dyssynchrony measured by all the dyssynchrony indices tended to be more prevalent in the patients with a wide QRS duration; however, no significant difference was found between the QRS groups. 

**Table 1:** Baseline characteristics and conventional echocardiographic measurements of the study population (N=106)*

Age (y)	60.5±12.1
Male gender	94 (88.7)
QRS duration (ms)	149.1±36.6
Left bundle branch block	31 (29.2)
Right bundle branch block	6 (5.7)
Diabetes mellitus	22 (20.8)
Atrial fibrillation	2 (1.9)
Pharmacological medication	
Loop diuretics	50 (47.2)
Spironolactone	79 (65.1)
ACEIs/ARBs	86 (81.1)
β-blockers	79 (74.5)
Calcium-blockers	24 (22.6)
Digoxin	33 (31.1)
Conventional Echocardiography	
Mitral regurgitation grade	1.7±0.8
Tricuspid regurgitation grade	1.3±0.8
Left ventricular ejection fraction (%)	22.3±6.7
Pulmonary artery pressure (mm Hg)	42.5±17.4
Systolic left ventricular dimension (mm)	57.1±15.2
Diastolic left ventricular dimension (mm)	65.2±8.8
Left ventricular end-diastolic volume (mL)	180.2±61.2
Left ventricular end-systolic volume (mL)	139.9±58.7

ACEIs, Angiotensin-converting enzyme inhibitors; ARBs, Angiotensin-receptor blockers

* Data are presented as means±SD or n (%).

Wall motion evaluation was technically feasible in 1 263 segments, from which 875 (69.3%) segments scored as normal or hypokinetic, 369 (29.2%) as akinetic, and 19 (1.5%) as aneurismal. 

**Figure 2:**
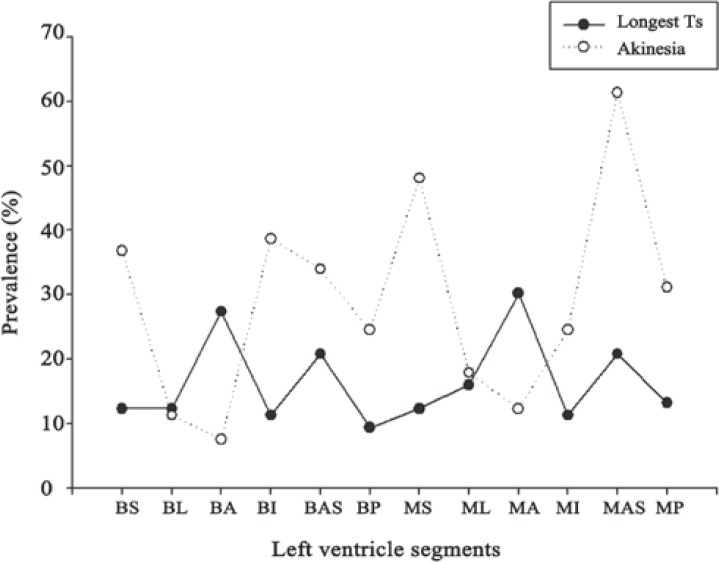
Frequencies of the latest activated regions and akinetic sites in 12 different left ventricular segments.

BS, Basal-septal; BL, Basal-lateral; BA, Basal-anterior; BI, Basal-inferior; BAS, Basal-anteroseptal; BP, Basal-posterior; MS, Mid-septal; ML, Mid-lateral; MA, Mid-anterior; MI, Mid-basal inferior; MAS, Mid-basal anteroseptal; MP, Mid-basal posterior

The frequencies for the latest activated and akinetic sites within the different LV segments are depicted in Figure 2. The latest activation sites (with longest Ts) were most frequently located in the mid and basal anterior (32 [30.2%] and 29 [27.4%], respectively), whereas the most common sites of akinesia were the mid-anteroseptal segments in 65 (61.3%) and mid-septal segments in 51 (48.1%) cases. Table 3 shows the agreement levels between the regions with longest Ts and the presence of akinesia in the patients as a whole and also in each QRS group. Overall, there was no significant concordance between the presence of latest activation and akinesia either in all the patients (Φ = -0.050; p value = 0.075) or in the QRS groups (QRS duration ≤ 120 ms [Φ = -0.009; p value = 0.875] and QRS duration > 120 ms [Φ = -0.071; p value = 0.085]). Detailed analysis within the segments indicated a significant, but weak, agreement between akinesia and delayed activation in the basal-lateral segments (Φ = 0.229; p value = 0.018). This agreement was good in the subgroup of patients with QRS duration ≤ 120 ms (Φ = 0.707; p value < 0.001).

**Table 2:** Tissue Doppler imaging findings in the whole study population and comparisons between the QRS groups^*^

	Total (N=106)	QRS Duration (ms)		P value
		≤ 120 (n=27)	˃ 120 (n=79)	
Ts of the Basal Segments (ms)				
Base-septal	169.4±56.3	149.1±34.2	178.9±58.2	0.014
Base-lateral	176.6±60.6	146.5±52.7	187.0±60.0	0.002
Base-anterior	161.6±50.8	146.3±51.5	166.8±61.9	0.124
Base-anteroseptal	161.0±52.4	153.1±42.2	165.7±52.5	0.269
Base-inferior	185.2±59.2	169.6±66.8	191.8±53.0	0.083
Base-posterior	191.4±66.6	169.3±65.4	202.7±59.2	0.016
Ts of the Middle Segments (ms)				
Mid-septal	172.6±52.5	150.0±33.4	180.4±55.7	0.001
Mid-lateral	168.3±54.9	146.5±52.1	175.8±54.1	0.016
Mid-anterior	159.3±57.8	133.5±52.8	168.1±57.0	0.007
Mid-anteroseptal	165.9±50.7	153.3±43.5	170.2±52.5	0.135
Mid-inferior	190.3±60.3	170.4±68.2	197.2±56.1	0.022
Mid-posterior	188.5±64.5	163.9±68.7	197.0±61.2	0.010
Interventricular Dyssynchrony Index				
Interventricular mechanical delay (ms)	33.4±24.6	19.0±13.4	38.3±25.7	< 0.001
Interventricular dyssynchrony (cut-off = 40)	33 (31.1)	3 (11.1)	30 (38.0)	0.009
Intraventricular Dyssynchrony Indices				
All segments delay (ms)	107.3±50.4	100.2±59.6	109.7±47.0	0.397
Dyssynchrony using all segments delay (cutoff = 105 ms)	52 (49.1)	10 (37.0)	42 (53.2)	0.148
All segments standard deviation (ms)	38.5±19.7	36.7±26.7	39.2±16.8	0.565
Dyssynchrony using all segments standard deviation (cutoff = 34.4 ms)	55 (51.9)	12 (44.4)	43 (54.4)	0.370
Basal segments delay (ms)	92.6±47.4	86.8±54.7	95.7±49.0	0.434
Dyssynchrony using basal segments delay (cutoff = 78 ms)	56 (53.8)	13 (48.1)	43 (55.8)	0.510
Basal segments standard deviation (ms)	39.7±22.8	36.7±29.1	40.8±20.3	0.423
Dyssynchrony using basal segments standard deviation (cutoff = 34.5 ms)	54 (50.9)	11 (40.7)	43 (54.4)	0.219

Continuous data are presented as means±SD or n(%).

Ts, Time to peak systolic velocity; ms, Milliseconds

**Table 3:** Evaluation of agreement between akinesia and latest activation site in various left ventricular (LV) segments.

LV segments	Φ coefficient		
	Total (N=106)	QRS ≤ 120 ms (n=27)	QRS > 120 ms (n=79)
Basal			
Septal	0.149	-0.235	-0.123
Lateral	0.229*	0.707**	0.009
Anterior	-0.015	0.135	-0.059
Inferior	-0.120	-0.120	-0.145
Anteroseptal	0.072	0.043	-0.110
Posterior	-0.116	0.329	0.033
Middle			
Septal	-0.072	0.027	-1.000
Lateral	-0.070	0.184	-0.140
Anterior	0.005	-0.042	0.023
Inferior	-0.079	-0.255	0.002
Anteroseptal	-0.023	-0.227	0.042
Posterior	-0.082	-0.043	-0.126
Total	-0.050	-0.009	-0.071

* P value = 0.018

** P value < 0.001

## Discussion

The results of the present study demonstrated that the latest LV activation segments were most frequently the mid and basal anterior segments, while the akinetic sites were predominantly located in the mid-septal and mid-anteroseptal segments. Overall, the akinetic segments were not matched with the most delayed activation regions.

Previous studies have reported a favorable response following lead positioning in the most delayed LV activated site/s.^12^^, ^^13^ Based on our results, there was no concordance between the akinetic segments and the regions with latest mechanical activation. Thus, these akinetic areas may not appear to be ideal site/s for pacing. In this regard, Arzola-Castaner et al.^   28 ^ also reported no significant impact on the CRT outcome following lead positioned in proximity to akinetic segments. It should be noted that akinetic segments cannot be regarded exactly as infracted regions. Akinesia is a sensitive, but not specific, indicator of transmural infarction. While 100% of the pathologically transmural infracted segments are akinetic or dyskinetic, 8% of the akinetic or dyskinetic regions are histologically normal and 29% are subendocardially infracted zones.^   18 ^ Therefore, it is not correct to generalize our result to the infracted segments. Future studies using contrast-enhanced magnetic resonance imaging or myocardial contrast echocardiography^16^^, ^^29^ would help investigators to better evaluate the extent of transmural scar, myocardial viability, and association between mechanical activation timing and infracted segments. 

The variable forms of LV activation and the heterogeneous pattern of systolic asynchrony in patients with severe heart failure, particularly in those with ischemic cardiomyopathy,^30^^, ^^31^ may explain a possible mismatch between the akinetic segments and the latest activated sites in patients with heart failure. Currently, there are several noninvasive imaging techniques such as 3-dimensional noncontact LV endocardial mapping and circumferential and longitudinal strain imaging which could help investigators to have better estimation on LV dyssynchrony patterns in the presence of akinesia by providing the exact characterization of the LV activation sequence.^31^^, ^^32^ 

Given the significant relationship between the QRS duration and regional Ts, we re-analyzed the data according to the QRS groups. The basal-lateral segments in the patients with a narrow QRS duration constituted the only site in which akinesia and latest activation showed a good agreement. Future studies applying more precise methods are needed to explain or even confirm the finding. 

This study suffers from some limitations. 2D echocardiography appears to tend to overestimate the extent of dyssynergic areas^18^^, ^^33^ because of its low ability to discriminate between the infracted and adjacent zones via an evaluation of wall motion abnormalities. Therefore, it is likely that the determined akinetic areas are not exclusively confined to the infracted zones. Furthermore, lack of any data on CRT long-term results hindered us from further analysis on association between the sites of lead placement and the CRT outcome. In addition, limitations of TDI such as measuring only the vector of motion that is parallel to the direction of the ultrasound beam and measuring absolute tissue velocity which makes it unable to discriminate passive motion (related tethering) from active motion (fiber shortening or lengthening) may affect the results.

## Conclusion

The akinetic segments on 2D echocardiogram were not matched with the area of maximal delay to peak velocity on TDI in our patients with the ischemic type of heart failure. Further studies are needed to elucidate the association between LV infracted regions and the latest activated sites in patients with the ischemic type of cardiomyopathy. 

## Notes:

***This paper should be cited as:*** Sadeghian H, Kousari A, Majidi S, Rezvanfard M, Kazemisaeid A, Moezzi SA, Vasheghani Farahani A, Abdar Esfahani M, Sahebjam M, Zoroufian A, Sadeghian A. Association between Latest Activated Sites in the Left Ventricle and Akinetic Segments in Patients with Ischemic Cardiomyopathy. J Teh Univ Heart Ctr 2016;11(3):115-122.

## References

[1] Dickstein K, Cohen-Solal A, Filippatos G, McMurray JJ, Ponikowski P, Poole-Wilson PA, Strömberg A, van Veldhuisen DJ, Atar D, Hoes AW, Keren A, Mebazaa A, Nieminen M, Priori SG, Swedberg K (2008). ESC Guidelines for the diagnosis and treatment of acute and chronic heart failure 2008: the Task Force for the Diagnosis and Treatment of Acute and Chronic Heart Failure 2008 of the European Society of Cardiology Developed in collaboration with the Heart Failure Association of the ESC (HFA) and endorsed by the European Society of Intensive Care Medicine (ESICM). Eur Heart J.

[2] Yu CM, Lin H, Zhang Q, Sanderson JE (2003). High prevalence of left ventricular systolic and diastolic asynchrony in patients with congestive heart failure and normal QRS duration. Heart.

[3] Ghio S, Constantin C, Klersy C, Serio A, Fontana A, Campana C, Tavazzi L (2004). Interventricular and intraventricular dyssynchrony are common in heart failure patients, regardless of QRS duration. Eur Heart J.

[4] Buch E, Lellouche N, De Diego C, Vaseghi M, Cesario DA, Fujimura O, Wiener I, Child JS, Boyle NG, Shivkumar K (2007). Left ventricular apical wall motion abnormality is associated with lack of response to cardiac resynchronization therapy in patients with ischemic cardiomyopathy. Heart Rhythm.

[5] Pavlopoulos H, Nihoyannopoulos P (2010). Recent advances in cardiac resynchronization therapy: echocardiographic modalities, patient selection, optimization, non-responders--all you need to know for more efficient CRT. Int J Cardiovasc Imaging.

[6] Abraham WT (2006). Cardiac resynchronization therapy. Prog Cardiovasc Dis.

[7] Bank AJ, Kelly AS (2006). Tissue Doppler imaging and left ventricular dyssynchrony in heart failure. J Card Fail.

[8] Bax JJ, Molhoek SG, van Erven L, Voogd PJ, Somer S, Boersma E, Steendijk P, Schalij MJ, Van der Wall EE (2003). Usefulness of myocardial tissue Doppler echocardiography to evaluate left ventricular dyssynchrony before and after biventricular pacing in patients with idiopathic dilated cardiomyopathy. Am J Cardiol.

[9] Ansalone G, Giannantoni P, Ricci R, Trambaiolo P, Laurenti A, Fedele F, Santini M (2001). Doppler myocardial imaging in patients with heart failure receiving biventricular pacing treatment. Am Heart J.

[10] Knebel F, Reibis RK, Bondke HJ, Witte J, Walde T, Eddicks S, Baumann G, Borges AC (2004). Tissue Doppler echocardiography and biventricular pacing in heart failure: patient selection, procedural guidance, follow-up, quantification of success. Cardiovasc Ultrasound.

[11] Murphy RT, Sigurdsson G, Mulamalla S, Agler D, Popovic ZB, Starling RC, Wilkoff BL, Thomas JD, Grimm RA (2006). Tissue synchronization imaging and optimal left ventricular pacing site in cardiac resynchronization therapy. Am J Cardiol.

[12] Van de Veire NR, Marsan NA, Schuijf JD, Bleeker GB, Wijffels MC, van Erven L, Holman ER, De Sutter J, van der Wall EE, Schalij MJ, Bax JJ (2008). Noninvasive imaging of cardiac venous anatomy with 64-slice multi-slice computed tomography and noninvasive assessment of left ventricular dyssynchrony by 3-dimensional tissue synchronization imaging in patients with heart failure scheduled for cardiac resynchronization therapy. Am J Cardiol.

[13] Ansalone G, Giannantoni P, Ricci R, Trambaiolo P, Fedele F, Santini M (2002). Doppler myocardial imaging to evaluate the effectiveness of pacing sites in patients receiving biventricular pacing. J Am Coll Cardiol.

[14] Fukuda K, Oki T, Tabata T, Iuchi A, Ito S (1998). Regional left ventricular wall motion abnormalities in myocardial infarction and mitral annular descent velocities studied with pulsed tissue Doppler imaging. J Am Soc Echocardiogr.

[15] Lambiase PD, Rinaldi A, Hauck J, Mobb M, Elliott D, Mohammad S, Gill JS, Bucknall CA (2004). Non-contact left ventricular endocardial mapping in cardiac resynchronisation therapy. Heart.

[16] Bleeker GB, Kaandorp TAM, Lamb HJ, Boersma E, Steendijk P, de Roos A, van der Wall EE, Schalij MJ, Bax JJ (2006). Effect of posterolateral scar tissue on clinical and echocardiographic improvement after cardiac resynchronization therapy. Circulation.

[17] Edner M, Ring M, Särev T (2010). Sequential biventricular pacing improves regional contractility, longitudinal function and dyssynchrony in patients with heart failure and prolonged QRS. Cardiovasc Ultrasound.

[18] Weiss JL, Bulkley BH, Hutchins GM, Mason SJ (1981). Two-dimensional echocardiographic recognition of myocardial injury in man: comparison with postmortem studies. Circulation.

[19] Sadeghian H, Majd-Ardakani J, Lotfi-Tokaldany M (2009). Assessment of myocardial viability: selection of patients for viability study and revascularization. J Teh Univ Heart Ctr.

[20] Gorcsan J, 3rd, Abraham T, Agler DA, Bax JJ, Derumeaux G, Grimm RA, Martin R, Steinberg JS, Sutton MS, Yu CM (2008). American Society of Echocardiography for cardiac resynchronization therapy: recommendations for performance and reporting--a report from the American Society of Echocardiography Dyssynchrony Writing Group endorsed by the Heart Rhythm Society. J Am Soc Echocardiogr.

[21] Feigenbaum H, Armstrong WF, Ryan T, eds (2005). Feigenbaum’s Echocardiography.

[22] Feigenbaum H, Armstrong WF, Ryan T, eds (2005). Feigenbaum’s Echocardiography.

[23] Niu H, Hua W, Zhang S, Sun X, Wang F, Chen K, Chen X (2007). Prevalence of dyssynchrony derived from echocardiographic criteria in heart failure patients with normal or prolonged QRS duration. Echocardiography.

[24] Sadeghian H, Salehi R, Lotfi-Tokaldany M, Fallah N, Abbasi SH (2007). Accuracy of dobutamine stress echocardiography in detecting recovery of contractile reserve after revascularization of ischemic myocardium. J Teh Univ Heart Ctr.

[25] Borges AC, Kivelitz D, Walde T, Reibis RK, Grohmann A, Panda A, Wernecke KD, Rutsch W, Hamm B, Baumann G (2003). Apical tissue tracking echocardiography for characterization of regional left ventricular function: comparison with magnetic resonance imaging in patients after myocardial infarction. J Am Soc Echocardiogr.

[26] Sadeghian H, Ahmadi F, Lotfi-Tokaldany M, Kazemisaeid A, Fathollahi MS, Goodarzynejad H (2010). Ventricular asynchrony of time-to-peak systolic velocity in structurally normal heart by tissue Doppler imaging. Echocardiography.

[27] Yu CM, Zhang Q, Fung JWH, Chan HCK, Chan YS, Yip GWK, Kong SL, Lin H, Zhang Y, Sanderson JE (2005). A novel tool to assess systolic asynchrony and identify responders of cardiac resynchronization therapy by tissue synchronization imaging. J Am Coll Cardiol.

[28] Arzola Castaner D, Taub C, Kevin Heist E, Fan D, Haelewyn K, Mela T, Picard MH, Ruskin JN, Singh JP (2006). Left ventricular lead proximity to an akinetic segment and impact on outcome of cardiac resynchronization therapy. J Cardiovasc Electrophysiol.

[29] Hummel JP, Lindner JR, Belcik JT, Ferguson JD, Mangrum JM, Bergin JD, Haines DE, Lake DE, DiMarco JP, Mounsey JP (2005). Extent of myocardial viability predicts response to biventricular pacing in ischemic cardiomyopathy. Heart Rhythm.

[30] Sogaard P, Egeblad H, Pedersen AK, Kim WY, Kristensen BO, Hansen PS, Mortensen PT (2002). Sequential versus simultaneous biventricular resynchronization for severe heart failure: Evaluation by tissue Doppler imaging. Circulation.

[31] Fung JWH, Yu CM, Yip G, Zhang Y, Chan H, Kum CC, Sanderson JE (2004). Variable left ventricular activation pattern in patients with heart failure and left bundle branch block. Heart.

[32] Helm RH, Leclercq C, Faris OP, Ozturk C, McVeigh E, Lardo AC, Kass DA (2005). Cardiac dyssynchrony analysis using circumferential versus longitudinal strain: implications for assessing cardiac resynchronization. Circulation.

[33] Lieberman AN, Weiss JL, Jugdutt BI, Becker LC, Bulkley BH, Garrison JG, Hutchins GM, Kallman CA, Weisfeldt ML (1981). Two-dimensional echocardiography and infarct size: relationship of regional wall motion and thickening to the extent of myocardial infarction in the dog. Circulation.

